# Supplementary figures and images for: Dual roles of EGO-1 and RRF-1 in regulating germline exo-RNAi efficiency in Caenorhabditis elegans
Source: EMBO Rep. 2025 Aug 11;26(18):4503–31. doi: 10.1038/s44319-025-00543-0 (PMC12457629; doi:10.1038/s44319-025-00543-0)

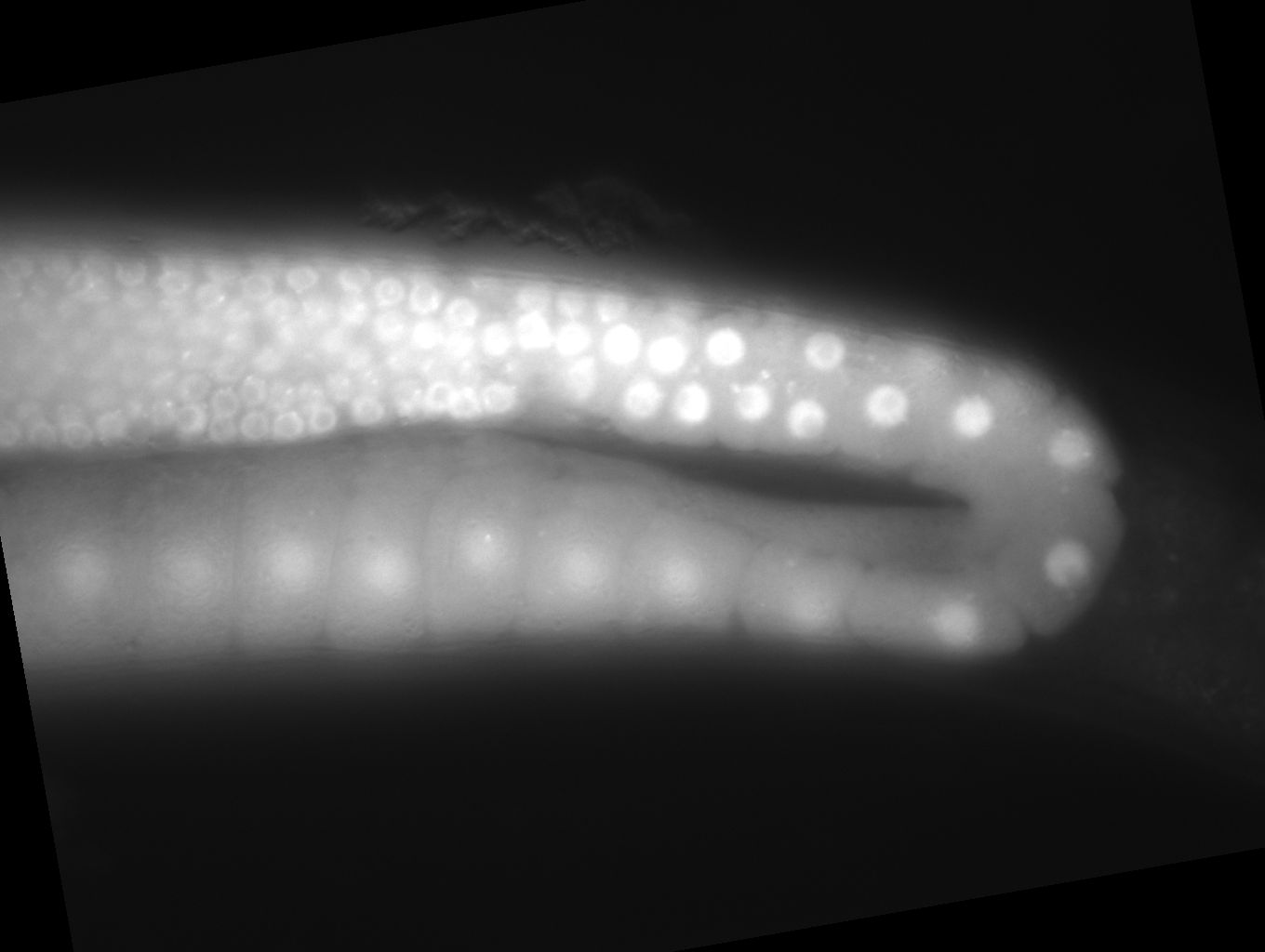

Supplement: Supplementary file 4 — Source data Fig. 3 [file 44319_2025_543_MOESM4_ESM.zip › figure3/3C/gfp_hrde1_S1198L.tif]

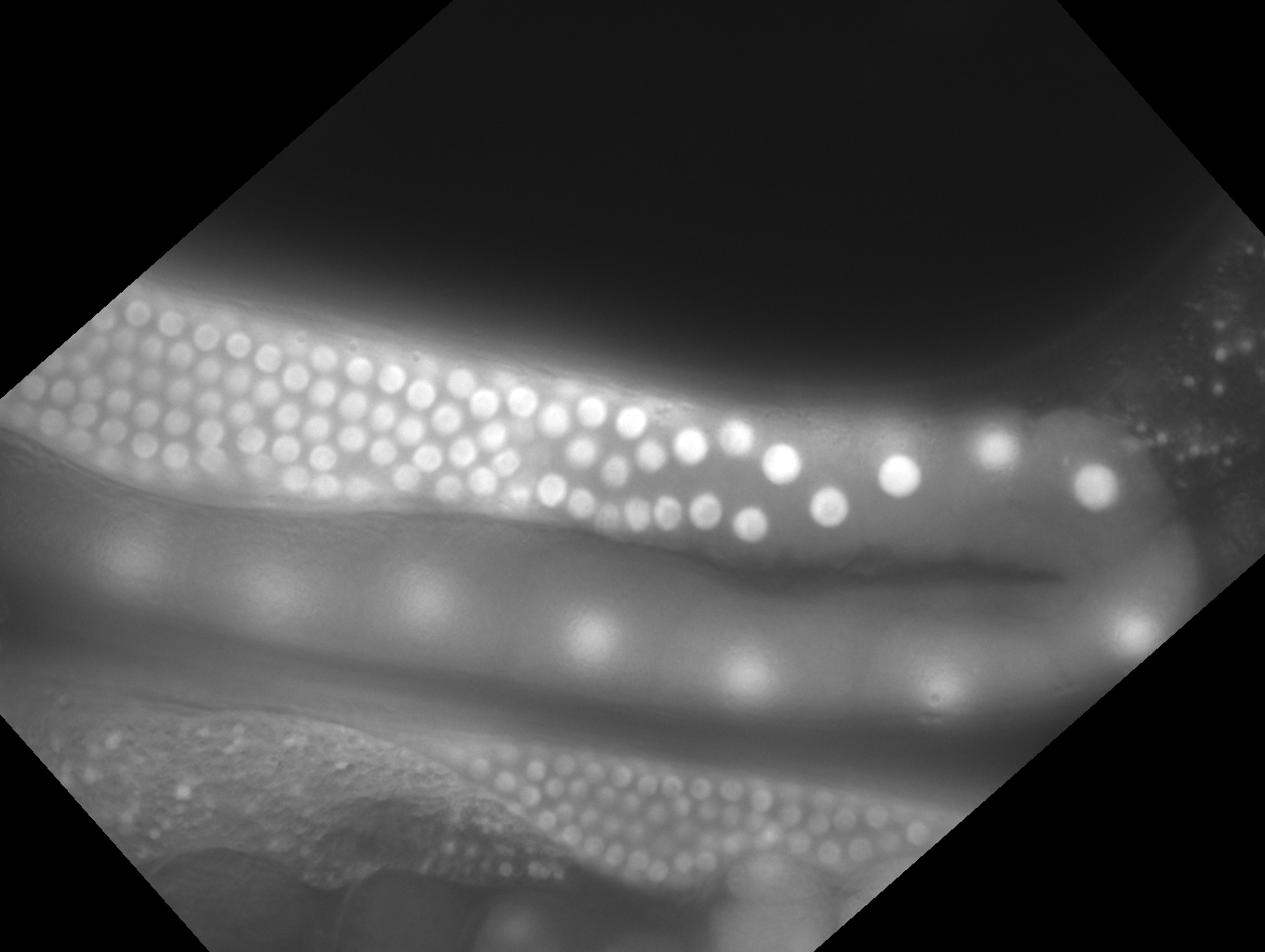

Supplement: Supplementary file 4 — Source data Fig. 3 [file 44319_2025_543_MOESM4_ESM.zip › figure3/3C/gfp_hrde1_wt.tif]

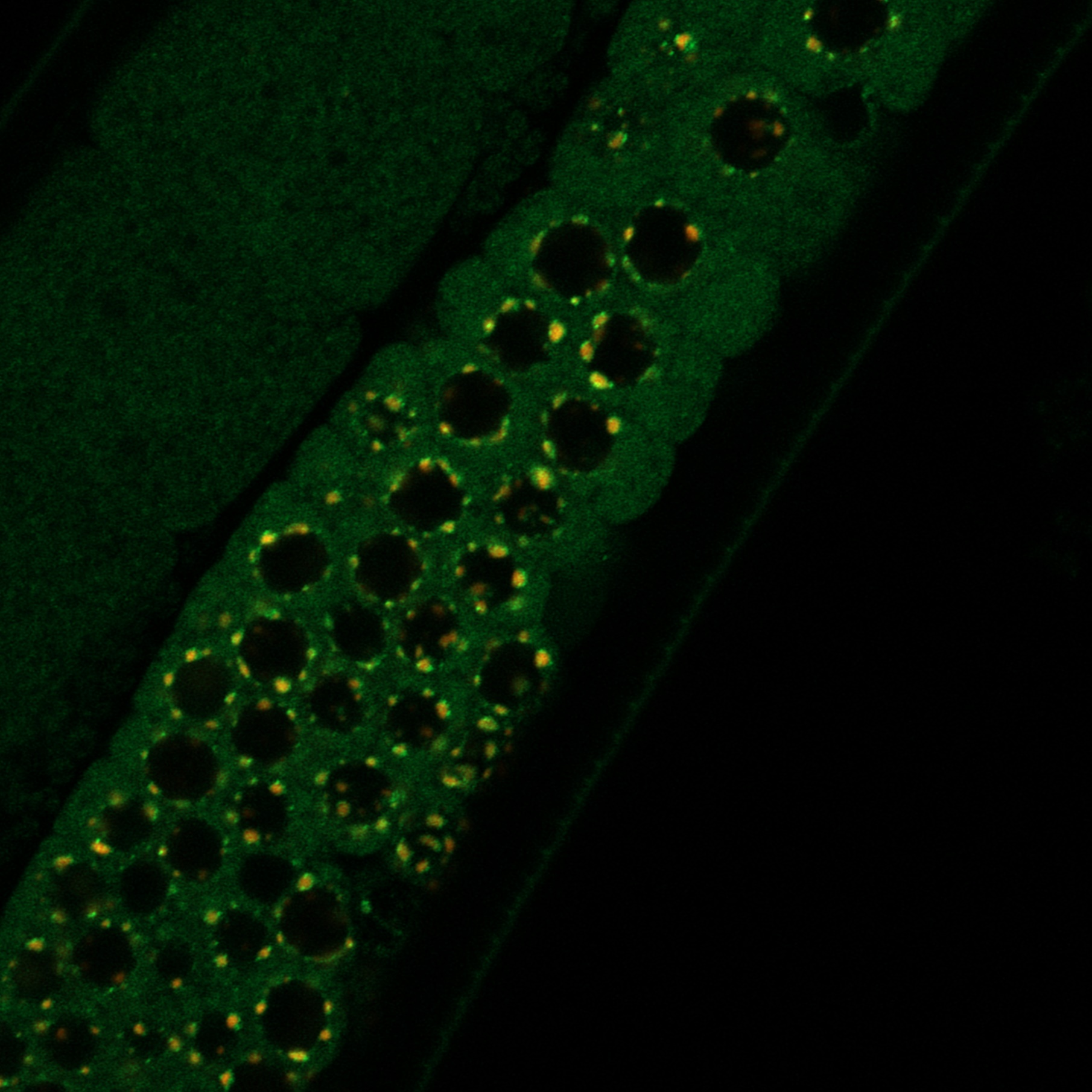

Supplement: Supplementary file 8 — Figure EVs, Tables Source Data [file 44319_2025_543_MOESM8_ESM.zip › figureEV3/EV3A/S1198L_source.tif]

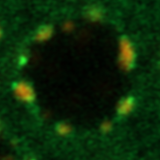

Supplement: Supplementary file 8 — Figure EVs, Tables Source Data [file 44319_2025_543_MOESM8_ESM.zip › figureEV3/EV3A/wt_cropped.tif]

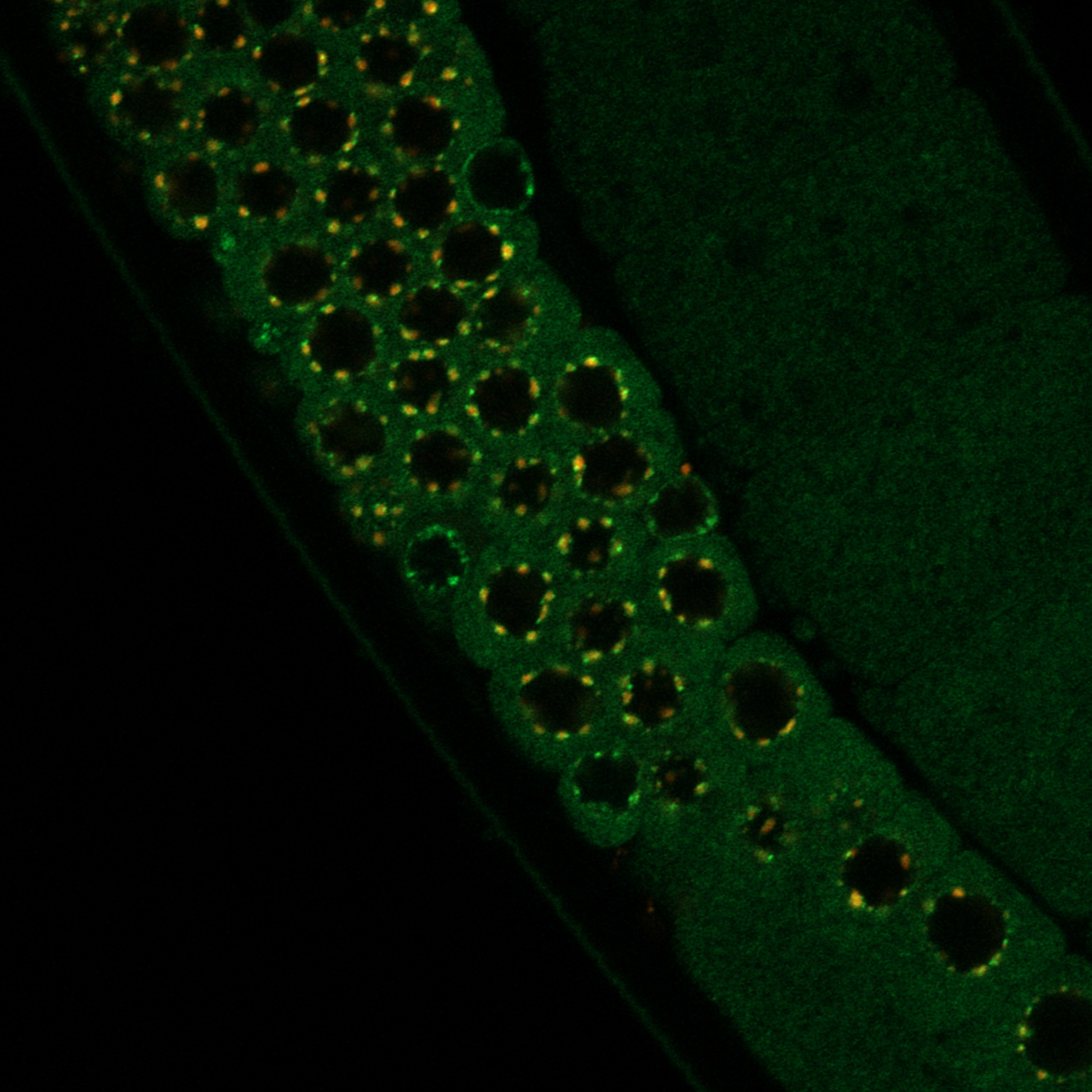

Supplement: Supplementary file 8 — Figure EVs, Tables Source Data [file 44319_2025_543_MOESM8_ESM.zip › figureEV3/EV3A/wt_source.tif]

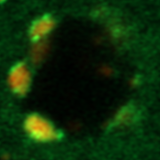

Supplement: Supplementary file 8 — Figure EVs, Tables Source Data [file 44319_2025_543_MOESM8_ESM.zip › figureEV3/EV3A/S1198L_cropped.tif]

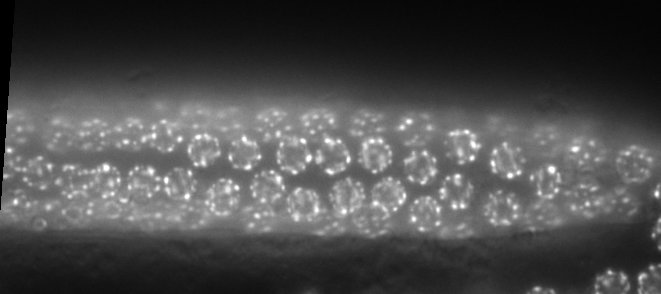

Supplement: Supplementary file 8 — Figure EVs, Tables Source Data [file 44319_2025_543_MOESM8_ESM.zip › figureEV4/EV4A/wt.tif]

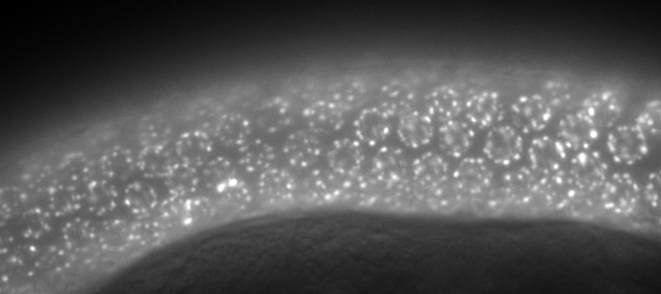

Supplement: Supplementary file 8 — Figure EVs, Tables Source Data [file 44319_2025_543_MOESM8_ESM.zip › figureEV4/EV4B/gk5320499.tif]
