# Supplementary material for: Dual roles of EGO-1 and RRF-1 in regulating germline exo-RNAi efficiency in Caenorhabditis elegans
Source: EMBO Rep. 2025 Aug 11;26(18):4503–31. doi: 10.1038/s44319-025-00543-0 (PMC12457629; doi:10.1038/s44319-025-00543-0)
Supplement: Supplementary file 9 — Expanded View Figures [file 44319_2025_543_MOESM9_ESM.pdf]

Expanded View Figures

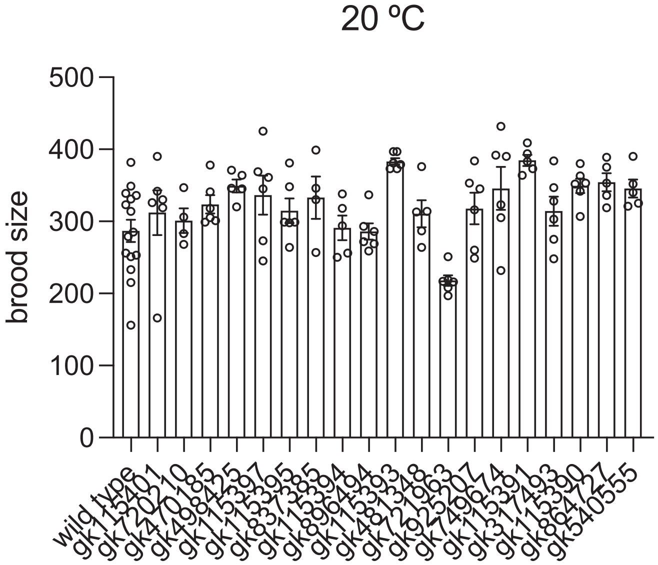

Figure EV1. ***ego-1*** **gk mutants show normal brood size.**

Brood size analysis of the indicated mutant animals that were cultured at 20 °C. The number of biological replicates (*n*) for each genotype was as follows: *gk115401*, *gk470185*, *gk115397*, *gk115395*, *gk896494*, *gk115393*, *gk721963*, *gk925207*, *gk749674*, *gk317493*, and *gk115390* ($n = 6$); *gk720210* and *gk837385* ($n = 4$); *gk498425*, *gk115394*, *gk481348*, *gk115391*, *gk864727*, and *gk540555* ($n = 5$). Error bars represent mean ± SEM. Wild-type data are shown for reference and are the same as those shown in Fig. 1D. Source data are available online for this figure.

 © The Author(s)

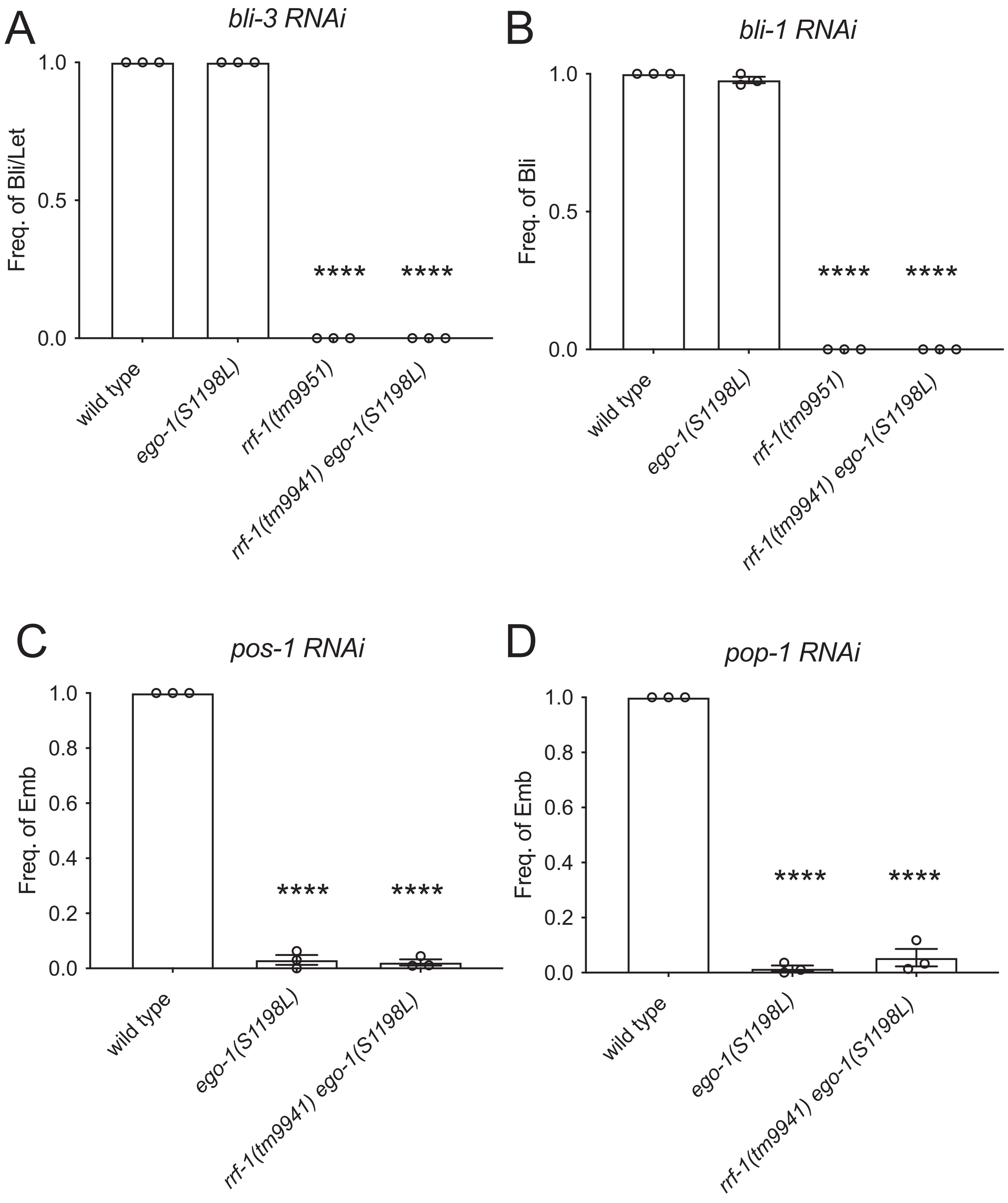

Figure EV2. *ego-1(S1198L)* is sensitive to somatic RNAi.

(**A**, **B**) Quantification of the frequency of the indicated mutant animals showing the expected phenotype under *bli-3* (**A**) and *bli-1* (**B**) feeding RNAi. Error bars represent mean ± SEM. Data represent values from three technical replicates. (**C**, **D**) Quantification of the frequency of indicated mutant animals showing the Emb phenotype under *pos-1* (**C**) and *pop-1* (**D**) feeding RNAi. Error bars represent mean ± SEM. Data represent values from three technical replicates. *P* values were determined using one-way ANOVA with Dunnett's multiple-comparisons test, compared to wildtype. **** indicates $p < 0.0001$. *P* values for specific comparisons were as follows: (**A**) *ego-1(S1198L)*, $p = 0.4044$; *rrf-1(tm9951)*, $p < 0.0001$; and *rrf-1(tm9941) ego-1(S1198L)*, $p < 0.0001$. (**B**) *ego-1(S1198L)*, $p = 0.0689$; *rrf-1(tm9951)*, $p < 0.0001$; and *rrf-1(tm9941) ego-1(S1198L)*, $p < 0.0001$. (**C**) *ego-1(S1198L)*, $p < 0.0001$; *rrf-1(tm9941) ego-1(S1198L)*, $p < 0.0001$. (**D**) *ego-1(S1198L)*, $p < 0.0001$; and *rrf-1(tm9941) ego-1(S1198L)*, $p < 0.0001$. Source data are available online for this figure.

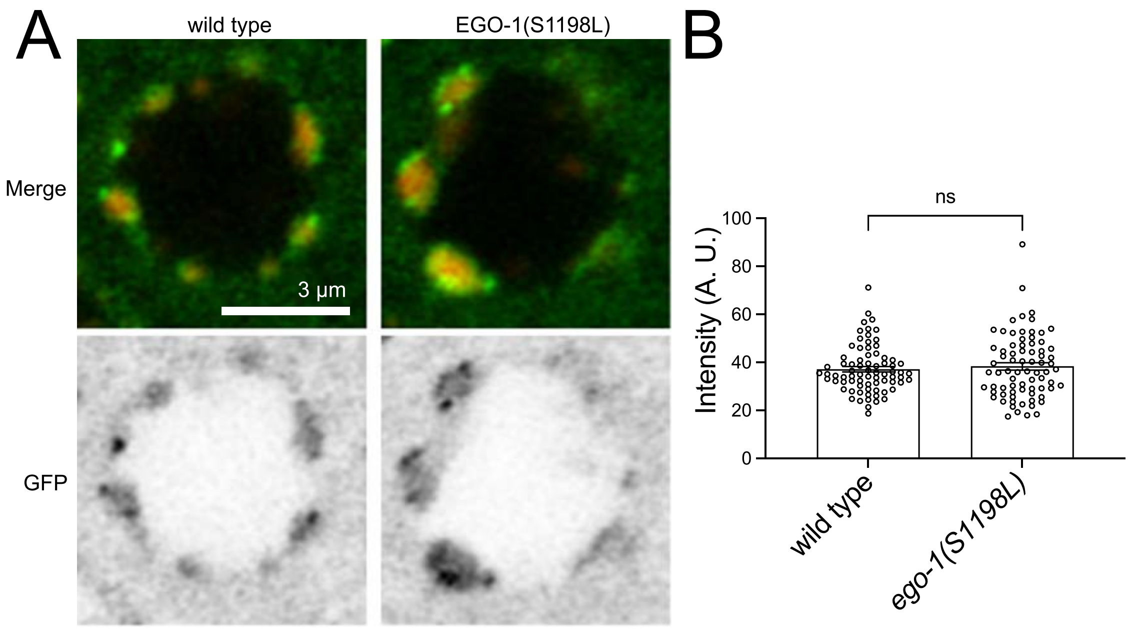

Figure EV3. GFP::EGO-1(S1198L) shows similar expression to that of GFP::EGO-1.

(**A**) Super-resolution image of a single pachytene nucleus of wild-type EGO-1 and EGO-1(S1198L) tagged with GFP (green) in late pachytene cells of 1-day-old adults. tagRFP::PGL-1 was used as a germ granule marker (red). (**B**) Bar graph shows the quantification of granular GFP::EGO-1 and GFP::EGO-1(S1198L) fluorescence signal intensity, with $n = 75$ for each genotype (25 animals, three ROIs (Nucleus and surrounded region) each). *P* values were determined using a Student's *t*-test. ns indicates not significant. Source data are available online for this figure.

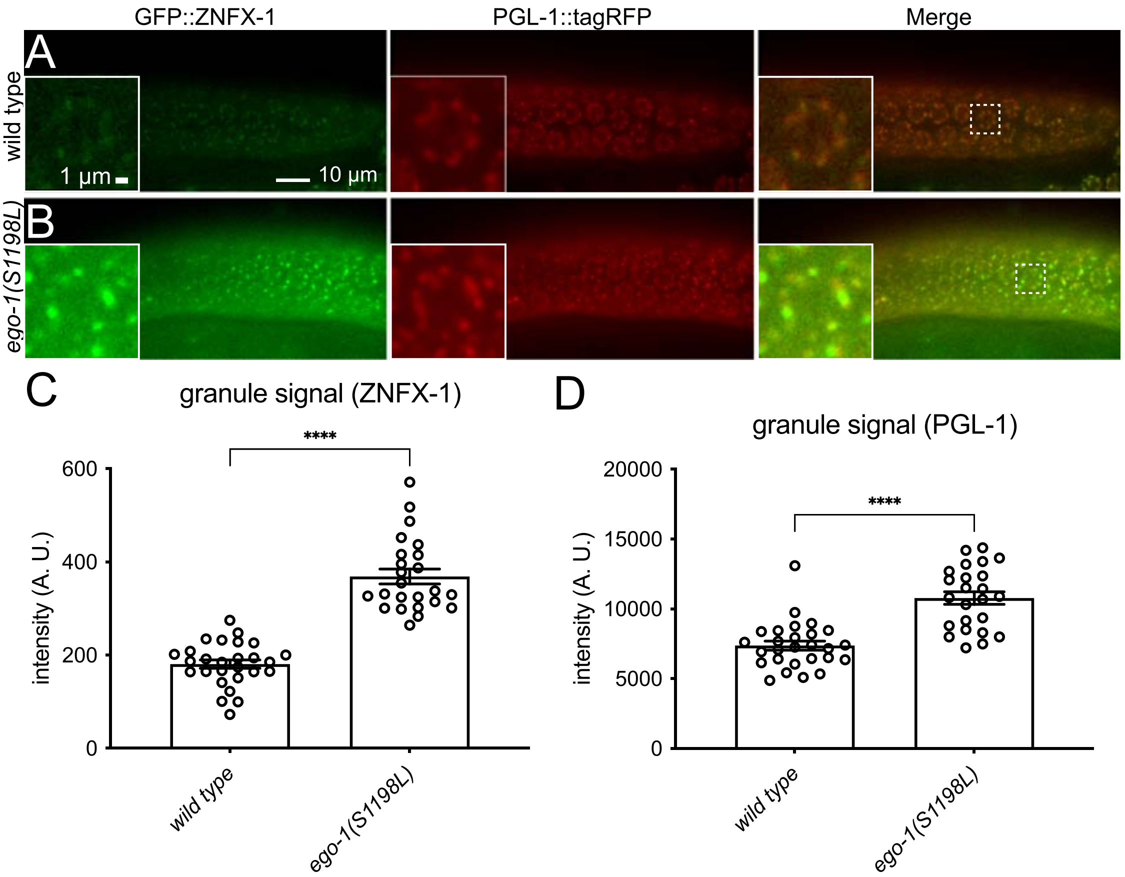

Figure EV4. Expression of GFP::ZNFX-1 and PGL-1::tagRFP in *ego-1(S1198L)*.

(**A**, **B**) Fluorescence images of germ cells showing the localization of GFP::ZNFX-1 and PGL-1::tagRFP in the wild-type (**A**) and *ego-1(S1198L)* (**B**). (**C**, **D**) Bar graphs show the quantification of granular GFP::ZNFX-1 (**C**) and PGL-1::tagRFP (**D**) fluorescence signal intensity. Error bars represent mean ± SEM. Figure EV4C shows data for wildtype ($n = 27$) and *ego-1(S1198L)* ($n = 24$) animals. Figure EV4D shows data for wildtype ($n = 24$) and *ego-1(S1198L)* ($n = 21$) animals. Statistical significance was determined using two-tailed unpaired *t*-tests. In all cases, the difference was significant (****$p < 0.0001$). Source data are available online for this figure.

© The Author(s)

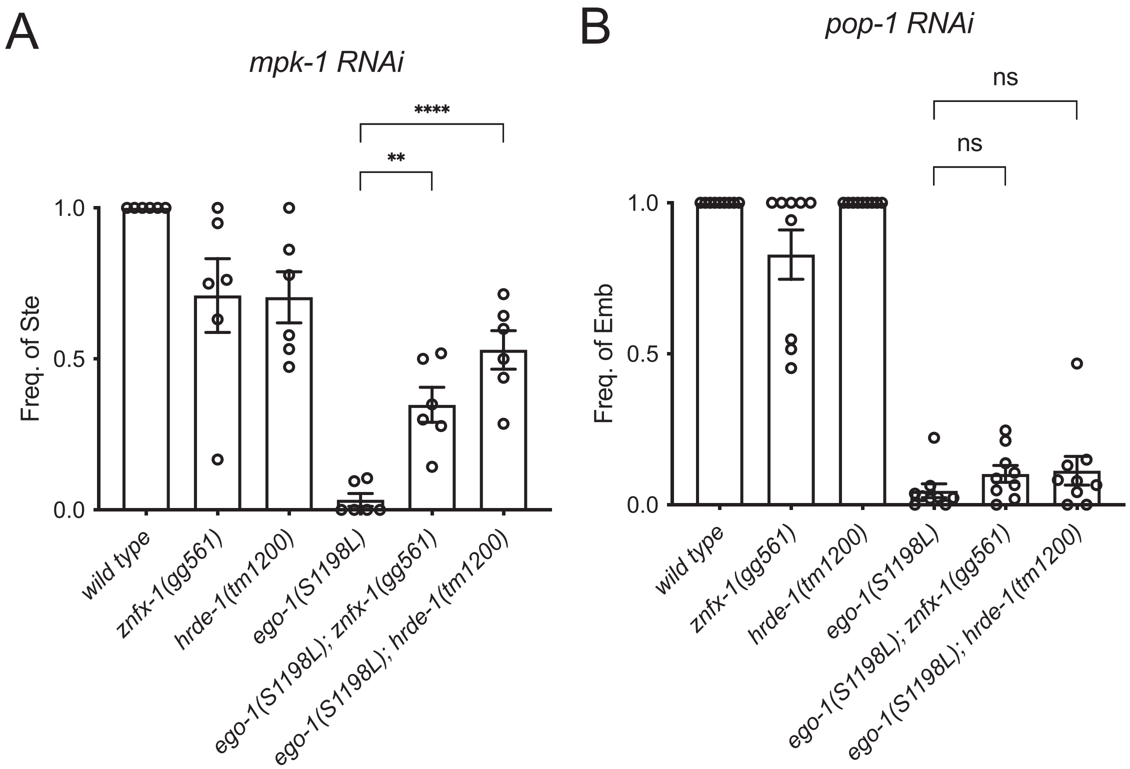

Figure EV5. Defects in germline exo-RNAi targeting *mpk-1* in *ego-1(S1198L)* are suppressed by *hrde-1* and *znfx-1* mutations.

(**A**) Quantification of the frequency of indicated mutant animals showing the Ste phenotype under *mpk-1* feeding RNAi. Error bars represent mean ± SEM. Data represent values from six technical replicates. *P* values were determined using one-way ANOVA with Dunnett's multiple-comparisons test. **** and ** indicate *p* value < 0.001 and < 0.01 (0.0074). (**B**) Quantification of the frequency of indicated mutant animals showing the Emb phenotype under *pop-1* feeding RNAi. Error bars represent mean ± SEM. Data represent values from nine technical replicates. *P* values were determined using one-way ANOVA with Dunnett's multiple-comparisons test compared to wildtype. **** indicates *p* value < 0.001. $p < 0.0001$ for V1128E, R539Q, and S1198L. For C823Y, $p = 0.7426$. Source data are available online for this figure.

 © The Author(s)

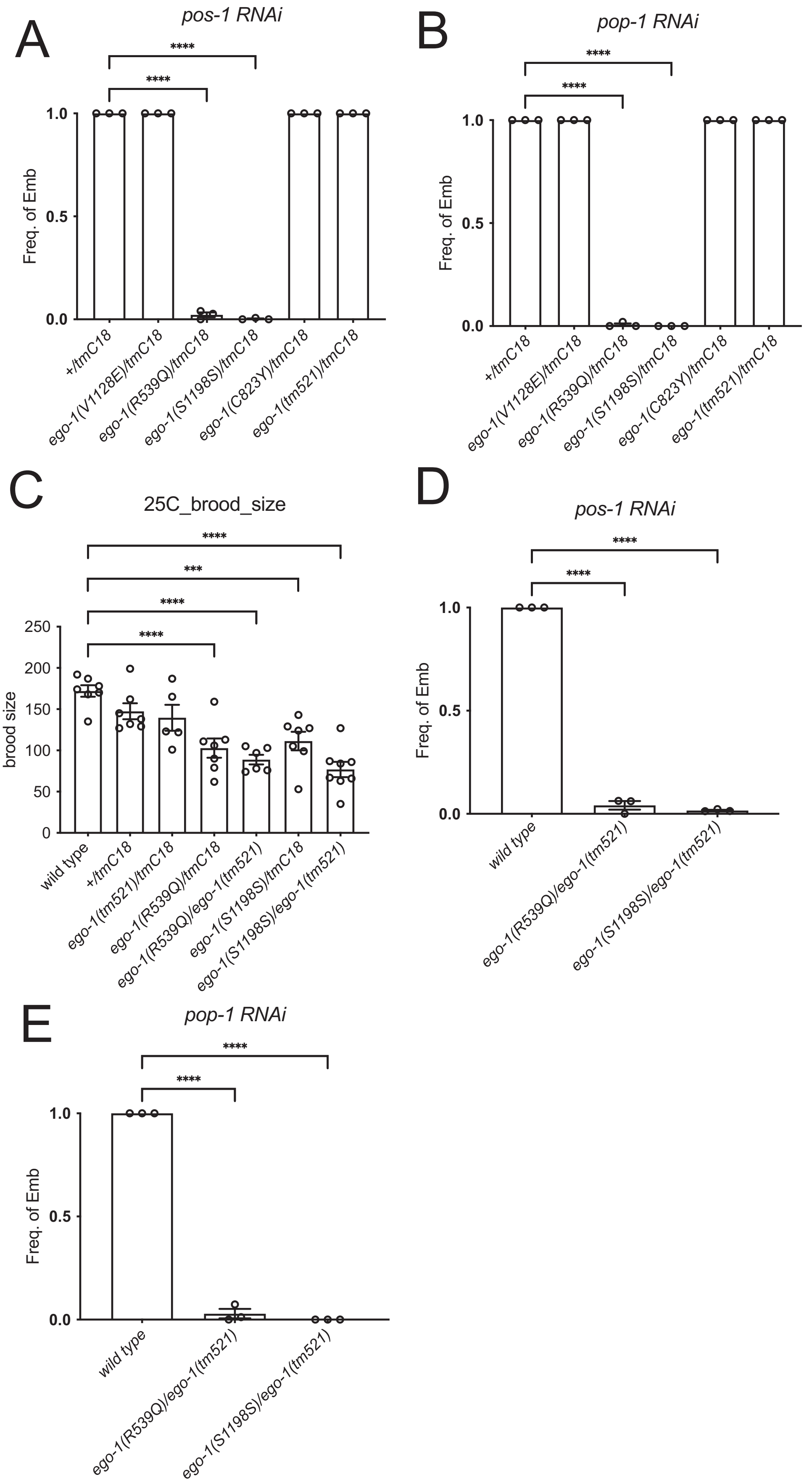
A
pos-1 RNAi
Freq. of Emb
+/tmC18
ego-1(V1128E)/tmC18
ego-1(R539Q)/tmC18
ego-1(S1198S)/tmC18
ego-1(C823Y)/tmC18
ego-1(tm521)/tmC18
B
pop-1 RNAi
Freq. of Emb
+/tmC18
ego-1(V1128E)/tmC18
ego-1(R539Q)/tmC18
ego-1(S1198S)/tmC18
ego-1(C823Y)/tmC18
ego-1(tm521)/tmC18
C
25C_brood_size
brood size
wild type
+/tmC18
ego-1(tm521)/tmC18
ego-1(R539Q)/tmC18
ego-1(R539Q)/ego-1(tm521)
ego-1(S1198S)/tmC18
ego-1(S1198S)/ego-1(tm521)
D
pos-1 RNAi
Freq. of Emb
wild type
ego-1(R539Q)/ego-1(tm521)
ego-1(S1198S)/ego-1(tm521)
E
pop-1 RNAi
Freq. of Emb
wild type
ego-1(R539Q)/ego-1(tm521)
ego-1(S1198S)/ego-1(tm521)

Figure EV6. RNAi-defective phenotype caused by *ego-1(R539Q)* and *ego-1(S1198L)* heterozygous animals.

(**A**) Quantification of the frequency of indicated mutant animals showing the Emb phenotype under *pos-1* feeding RNAi. Error bars represent mean ± SEM. Data represent values from three technical replicates. (**B**) Quantification of the frequency of indicated mutant animals showing the Emb phenotype under *pop-1* feeding RNAi. Error bars represent mean ± SEM. Data represent values from three technical replicates. In A and B, statistical analysis was performed using one-way ANOVA with Dunnett's multiple-comparisons test, compared to the +/tmC18 control. **** indicates $p < 0.0001$. (**A**) Significant differences were observed for *R539Q/tmC18* and *S1198L/tmC18* ($p < 0.0001$ for both). No significant differences were found for *V1128E/tmC18*, *C823Y/tmC18*, or *ego-1(tm521)/tmC18* ($p > 0.9999$ for all). (**B**) Significant differences were observed for *R539Q/tmC18* and *S1198L/tmC18* ($p < 0.0001$ for both). No significant differences were found for *V1128E/tmC18, C823Y/tmC18, or ego-1(tm521)/tmC18* ($p > 0.9999$ for all). (**C**) Brood size analysis of the indicated mutant animals that were cultured at 25 °C. Error bars represent mean ± SEM. Data represent values from wildtype ($n = 7$), *+/tmC18* ($n = 7$), *ego-1(tm521)/tmC18 (n = 5)*, *R539Q/tmC18* ($n = 7$), *R539Q/ego-1(tm521)* ($n = 6$), and *S1198L/tmC18* ($n = 7$). Statistical analysis was performed using one-way ANOVA with Dunnett's multiple-comparisons test, compared to wild-type control. *** indicates $p < 0.001$ and **** indicates $p < 0.0001$. Significant differences were observed for all *R539Q* and *S1198L* genotypes shown. No significant differences were found for *+/tmC18* or *ego-1(tm521)/tmC18*. (**D**) Quantification of the frequency of indicated mutant animals showing the Emb phenotype under *pos-1* feeding RNAi. Error bars represent mean ± SEM. Data represent values from three technical replicates. (**E**) Quantification of the frequency of indicated mutant animals showing the Emb phenotype under *pop-1* feeding RNAi. Error bars represent mean ± SEM. Data represent values from three technical replicates. In (**D**, **E**), statistical analysis was performed using one-way ANOVA with Dunnett's multiple-comparisons test, compared to wildtype. (**D**) *** indicates $p < 0.001$ and **** indicates $p < 0.0001$. Significant differences were observed for all *R539Q* and *S1198L* genotypes shown. No significant differences were found for *+/tmC18* or *ego-1(tm521)/tmC18*. (**E**) **** indicates $p < 0.0001$. *P* values for specific comparisons were as follows: *R539Q/ego-1(tm521)*, $p < 0.0001$; and *S1198L/ego-1(tm521)*, $p < 0.0001$. Source data are available online for this figure.

 © The Author(s)

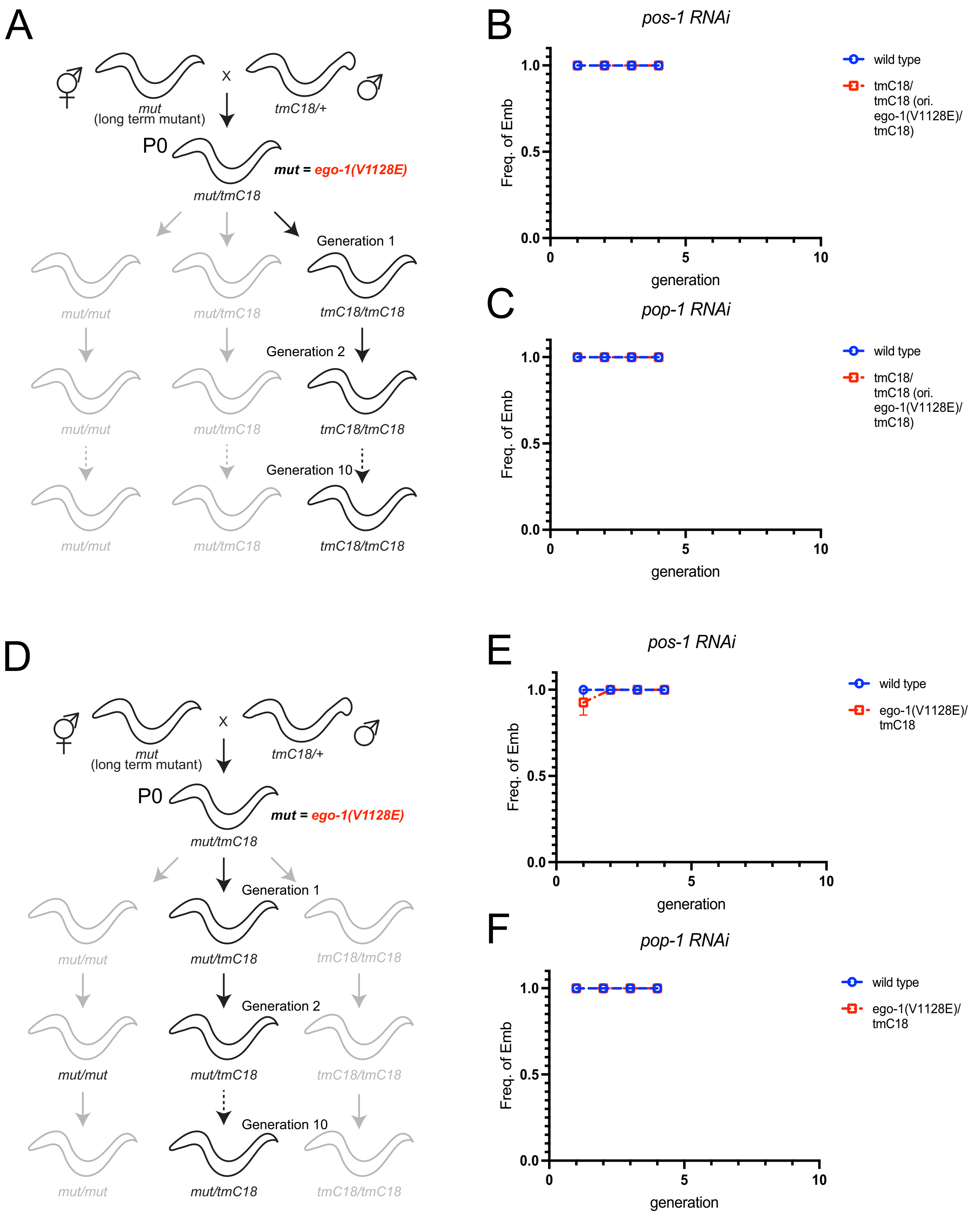
A
mut
(long term mutant)
x
tmC18/+
P0
mut = ego-1(V1128E)
mut/tmC18
Generation 1
mut/mut
mut/tmC18
tmC18/tmC18
Generation 2
mut/mut
mut/tmC18
tmC18/tmC18
Generation 10
mut/mut
mut/tmC18
tmC18/tmC18
B
pos-1 RNAi
Freq. of Emb
generation
wild type
tmC18/tmC18 (ori. ego-1(V1128E)/tmC18)
C
pop-1 RNAi
Freq. of Emb
generation
wild type
tmC18/tmC18 (ori. ego-1(V1128E)/tmC18)
D
mut
(long term mutant)
x
tmC18/+
P0
mut = ego-1(V1128E)
mut/tmC18
Generation 1
mut/mut
mut/tmC18
tmC18/tmC18
Generation 2
mut/mut
mut/tmC18
tmC18/tmC18
Generation 10
mut/mut
mut/tmC18
tmC18/tmC18
E
pos-1 RNAi
Freq. of Emb
generation
wild type
ego-1(V1128E)/tmC18
F
pop-1 RNAi
Freq. of Emb
generation
wild type
ego-1(V1128E)/tmC18

Figure EV7. Transgenerational effects of *ego-1(V1128E)* on germline exo-RNAi.

(**A**, **D**). Schematics of genetic crosses. P0 is the first generation in which cross-progenies are selected. (**B**, **C**) Quantification of the frequency of the indicated mutant animals showing the Emb phenotype under *pos-1* (**B**) and *pop-1* (**C**) feeding RNAi in the indicated generation. Data represent values from four biological replicates. These experiments were conducted using a similar experimental setup as in Fig. 5. Source data are available online for this figure.

 © The Author(s)

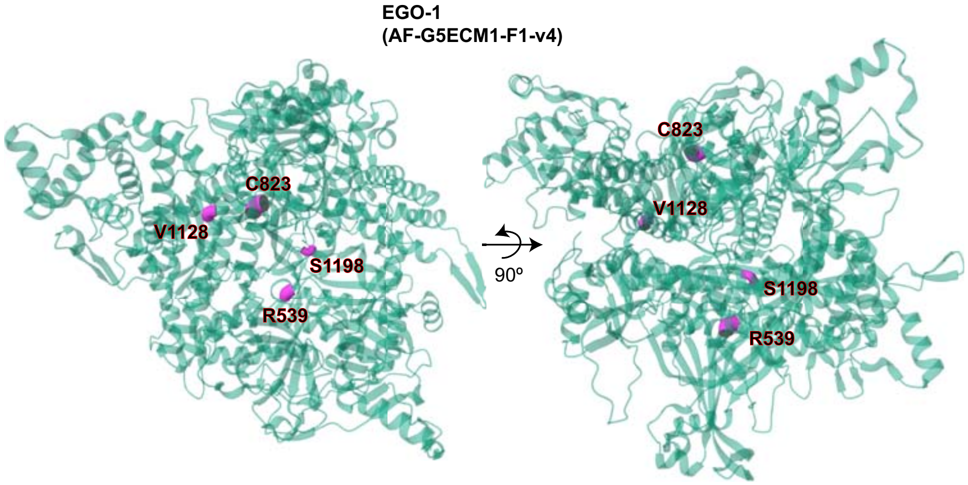

Figure EV8. Structure of wild-type EGO-1 with highlighted mutant sites (C623, V1128, R539, and S1198).

The 3D structure of the EGO-1 protein was predicted by Alphafold. Image was obtained from Uniprot.

© The Author(s)
